# The Importance of Supportive Leadership in Advancing Patient Safety

**DOI:** 10.7759/cureus.94056

**Published:** 2025-10-07

**Authors:** Tatsuya Fukami

**Affiliations:** 1 Patient Safety Division, Shimane University Hospital, Izumo, JPN

**Keywords:** healthcare teamwork, interprofessional collaboration in healthcare, leadership training, patient safety culture, psychological safety, safety leadership, supportive leadership, transformational leadership

## Abstract

Patient safety remains a core concern in healthcare systems worldwide. While technological innovations and clinical protocols are indispensable, the role of leadership in shaping safety culture has garnered increasing attention. Supportive leadership, a style emphasizing psychological safety, open communication, and empowerment, has emerged as a crucial determinant of safety outcomes. This narrative review synthesizes current evidence, theoretical frameworks, and lived experience to explore how supportive leadership contributes to patient safety. We argue that fostering supportive leadership at all organizational levels, and across professional roles, can lead to improved incident reporting, inter-professional collaboration, and systemic learning, ultimately reducing preventable harm and creating resilient health systems. Furthermore, we discuss how supportive leadership must also encompass cultural transformation, inclusive decision-making, and empowerment of not only healthcare professionals but also administrative staff, assistants, and security personnel who collectively form the frontline of patient care.

## Introduction and background

Patient safety, defined as the prevention of errors and adverse effects associated with healthcare, is one of the foundational goals of modern medicine [1]. Despite efforts over the past two decades, adverse events remain alarmingly common. The World Health Organization (WHO) estimates that one in 10 patients is harmed while receiving hospital care in high-income countries, with similar or higher rates in low- and middle-income settings [2]. Initially, the field of patient safety focused heavily on technical fixes, checklists, electronic health records, simulation-based training, and process redesign [3]. While these interventions are necessary, they are not sufficient. Increasingly, attention has turned toward the human and relational factors that underpin safety. Chief among these is leadership. Supportive leadership, broadly defined as a style that prioritizes trust, inclusion, empathy, and empowerment, is gaining traction as a central element in the advancement of patient safety [4]. Unlike authoritarian or transactional leadership, which emphasizes control, compliance, and performance metrics, supportive leadership aims to cultivate psychological safety, enable shared learning, and create resilient teams capable of adapting to complexity [5,6]. This review provides a comprehensive overview of the conceptual underpinnings, empirical support, and implementation strategies for supportive leadership. We argue that it is not merely a leadership “style” but a cultural foundation, one that should be purposefully cultivated within organizations if patient safety is to be truly realized [7].

## Review

Conceptual framework: What is supportive leadership?

Supportive leadership involves a deliberate orientation toward the needs, concerns, and development of others [8]. It emphasizes listening with empathy, validating individual contributions, facilitating open and transparent communication, and guiding teams without resorting to top-down control. Within healthcare, this model represents a shift away from rigid authority toward relational influence, enabling staff at all levels to participate meaningfully in decisions that affect their work and their patients [9]. In clinical settings, supportive leadership often manifests through visible presence, emotional availability, and responsiveness to staff concerns. Such leaders build trust by encouraging bidirectional feedback and acknowledging uncertainty as a normal feature of complex care [10]. In contrast to command-and-control leadership styles, supportive leadership thrives on humility, collective problem-solving, and continuous learning. This orientation not only strengthens professional relationships but also creates a psychological environment in which team members can innovate without fear of punitive consequences, a critical precondition for sustained patient safety improvement. These behaviors align closely with several well-established leadership frameworks. Transformational leadership seeks to inspire teams through a compelling vision and alignment of values [11]. Servant leadership focuses on putting the needs of team members first, supporting their growth and autonomy [12]. Authentic leadership values self-awareness, relational transparency, and a strong internal moral compass [13]. Inclusive leadership ensures that all voices, especially those often marginalized, are heard, valued, and incorporated into decision-making. Psychological safety, as defined by Edmondson, refers to the shared belief that it is safe to speak up, admit mistakes, and ask for help [14,15]. All these frameworks converge on the principle that leadership is most effective not when it dominates, but when it enables others to flourish. Supportive leadership, then, is not simply “being nice.” It is a deliberate practice of empowerment, clarity, and responsiveness that forms the foundation of resilient, high-functioning healthcare teams. By embedding these principles into daily routines, through consistent behavior, transparent decision-making, and equitable recognition, leaders can ensure that supportive leadership is not an abstract ideal but a lived reality within their organizations.

Supportive leadership and patient safety: empirical evidence

This section synthesizes findings from peer-reviewed studies, safety culture surveys, and real-world case analyses identified through a targeted literature search of PubMed, Scopus, and organizational reports from 2000-2024, using keywords such as “supportive leadership”, “patient safety”, “psychological safety”, and “healthcare leadership". The impact of leadership on patient safety has been well documented across a range of healthcare settings and cultures. In particular, supportive leadership has been linked to several dimensions of safety culture, including openness, teamwork, trust in management, and willingness to report concerns [15]. These domains, while intangible, directly influence the occurrence, reporting, and learning from medical errors. Evidence from the Agency for Healthcare Research and Quality (AHRQ) Hospital Survey on Patient Safety Culture shows that hospitals scoring highly on leadership support for safety also tend to have fewer adverse events and better interdepartmental collaboration [16]. Pronovost and colleagues, in their landmark work on safety culture, emphasized that leadership commitment must be visible, sustained, and integrated into day-to-day operations to have any lasting impact [4]. Similarly, Singer et al. found that the perceived responsiveness of leadership to safety concerns was one of the strongest predictors of staff engagement with safety initiatives [8]. Meta-analytic evidence further indicates that leadership behaviors account for a significant proportion of variance in safety culture scores, suggesting that leadership style is not merely correlated with, but may causally influence, safety outcomes. Beyond surveys, real-world behavior provides further confirmation. Leaders who regularly round on units, engage with staff, and invite input are more likely to identify latent hazards early and foster a climate of mutual accountability. In contrast, leaders who remain distant, punitive, or indifferent to frontline challenges inadvertently create conditions in which staff hesitate to report or address safety issues. Supportive leadership thus serves as both a buffer and a bridge, buffering staff from the stress of unsafe conditions and bridging the gap between policy and practice [17]. The role of psychological safety has also been extensively studied. Edmondson’s research on clinical teams demonstrated that when staff feel safe to speak up, they report more errors and learn more quickly from them [6]. This dynamic, more reporting leading to better outcomes, may seem counterintuitive at first glance but reflects a deeper truth: that improvement begins with awareness, and awareness requires trust. A systematic review by Okuyama et al. reinforced these findings, identifying a lack of leadership support as one of the main barriers to speaking up about patient safety concerns [7]. Without such support, even the most sophisticated reporting systems will fail to capture the information needed to prevent harm. Supportive leadership also has indirect effects on safety through its influence on burnout and staff retention. Burnout, characterized by emotional exhaustion, cynicism, and reduced personal efficacy, is not just a workforce issue; it is a safety issue [18]. Multiple studies have shown that burnout correlates with higher error rates, decreased patient satisfaction, and lower organizational performance [19]. Shanafelt and colleagues found that perceived leadership behavior is one of the strongest predictors of physician well-being [5]. Leaders who validate the emotional labor of healthcare, who set realistic expectations, and who advocate for adequate resources play a key role in mitigating burnout. In doing so, they not only support individual clinicians but also preserve team stability and institutional memory, two critical ingredients for safe, reliable care.

Real-world applications: learning from success

**Figure 1:**
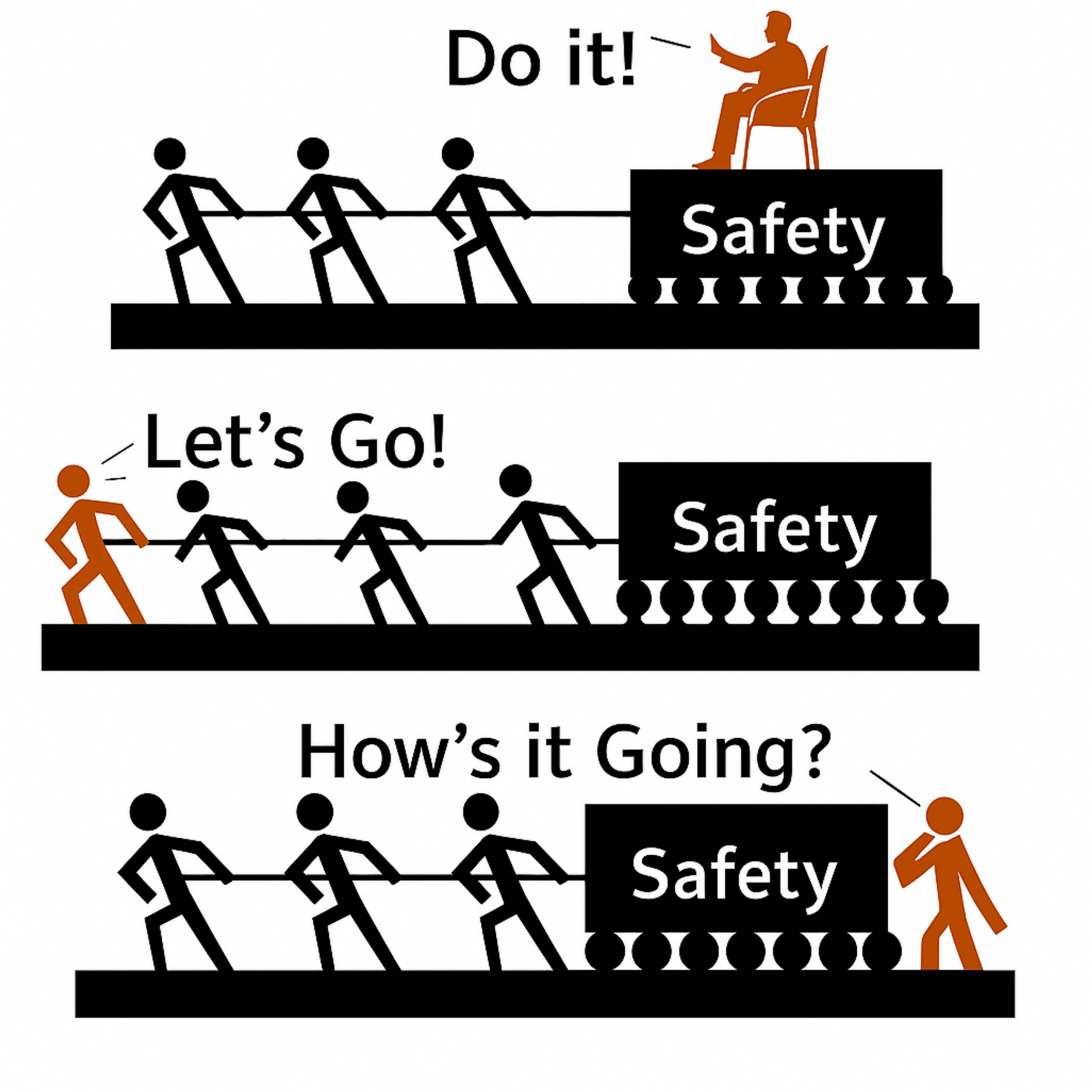
Three leadership styles in healthcare. Boss-type leaders command from above, captain-type leaders work alongside their team ("Let's Go!"), and follow-type leaders provide supportive feedback from behind ("How's it going?"). Each has different implications for patient safety and team dynamics. Illustration of three leadership styles in relation to patient safety: “Do it!” (boss style), “Let’s Go!” (captain style), and “How’s it going?” (follower style). This figure was created by the author based on general leadership typologies commonly used in healthcare safety training. No copyrighted materials were reused.

Several organizations have demonstrated the transformative power of supportive leadership in advancing patient safety. These case studies offer practical insight into how theory can be translated into action and sustained over time. At Virginia Mason Medical Center in Seattle, the commitment to safety was made explicit through the creation of the “Patient Safety Alert System” [20]. This system empowers any employee, regardless of role, to immediately halt a process if they believe patient safety is at risk. Such a radical flattening of authority is only possible within a culture of deep mutual trust, reinforced by leadership that prioritizes learning over blame. Senior executives round regularly, share data transparently, and model vulnerability when mistakes occur. Their approach demonstrates that supportive leadership is not about relinquishing responsibility, but about distributing it wisely. In the United Kingdom, the NHS Leadership Academy has placed inclusive and compassionate leadership at the center of its quality improvement strategy [21]. The “Leading for Safety” program explicitly trains leaders to support psychological safety, engage in active listening, and respond constructively to feedback. These competencies are evaluated through 360-degree reviews that include input from subordinates, peers, and patients. By embedding supportive leadership into training, assessment, and promotion pathways, the NHS signals its value not just rhetorically but structurally. In Japan, cultural norms of hierarchy and deference to authority have historically presented barriers to open communication. Recognizing this, several Japanese institutions have implemented programs such as TeamSTEPPS, which emphasize “captain-type” leadership, where senior staff do not merely supervise but participate actively alongside junior members [22]. This “Let’s Go!” approach redefines authority as responsibility and leadership as service. It encourages experienced clinicians to model expected behaviors, guide in real time, and create space for reflective dialogue. By situating leadership as a relational and developmental process rather than a static role, these programs help cultivate the kind of trust that is essential for patient safety (Figure 1).

Challenges and barriers

Despite its demonstrated value, supportive leadership faces significant challenges in healthcare environments. One of the most pervasive barriers is cultural resistance, particularly in systems characterized by rigid hierarchies and deeply entrenched traditions. In such settings, authority is often equated with infallibility, and questioning a superior, even with the best intentions, is viewed as insubordination [22]. This cultural dynamic inhibits open dialogue, reduces psychological safety, and ultimately jeopardizes patient care [23]. Another common obstacle is the lack of time and resources. Healthcare leaders often juggle multiple operational, financial, and regulatory responsibilities [24]. The daily pressures of managing schedules, budgets, and compliance can make it difficult to prioritize relationship-building or provide emotional support to staff. Yet, it is precisely in such high-stress environments that supportive leadership is most needed. Without intentional investment, however, it risks becoming an aspirational concept rather than a lived reality [25]. It is also important to recognize that even well-intentioned supportive leadership can be undermined if organizational policies or incentive structures conflict with its principles. For example, a leader may encourage open reporting of near misses, but if institutional metrics prioritize low incident numbers without context, staff may feel pressured to underreport. Measurement presents a further challenge. While clinical outcomes and financial metrics are relatively easy to quantify, leadership behaviors and their impact on safety culture are more difficult to assess. Traditional evaluation tools often fail to capture the nuances of trust, empathy, or psychological safety [26]. Without robust metrics, organizations may struggle to hold leaders accountable for these softer yet equally essential aspects of performance. Supportive leadership also cannot compensate for structural deficiencies. Under-resourced units, inadequate staffing, punitive regulatory regimes, and systemic inequities can overwhelm even the most committed leaders. While supportive leadership can mitigate the effects of these stressors, it cannot eliminate them alone [27]. Broader institutional reforms are necessary to create an environment in which supportive leadership can thrive and make a lasting impact on patient safety.

Future directions and recommendations

To embed supportive leadership into the fabric of healthcare, a multi-pronged strategy is required. First, leadership development must be democratized. Training should not be limited to senior executives but should extend to middle managers, team leads, and even students and residents. Everyone with influence over others, formally or informally, should be equipped with the skills and mindsets needed to lead supportively. Programs should focus on communication, emotional intelligence, conflict resolution, and coaching [28]. Second, organizations should implement 360-degree feedback systems that incorporate staff perceptions into leadership evaluation. These tools provide valuable insights into how leaders are experienced by those they lead, enabling personal growth and organizational alignment. Leaders should be rewarded not only for financial or operational outcomes but also for fostering safety culture, team engagement, and inclusivity [29]. In addition, research should aim to develop validated tools for measuring supportive leadership behaviors specific to healthcare contexts, enabling longitudinal assessment and benchmarking across institutions. These tools should integrate both quantitative (e.g., safety culture scores) and qualitative (e.g., narrative staff feedback) dimensions to capture the full scope of leadership impact. Third, incident reporting systems should be redesigned to reinforce supportive leadership. When a staff member reports an error or concern, the response they receive shapes their future behavior. Supportive leaders should acknowledge the courage it takes to speak up, provide non-punitive feedback, and demonstrate how the information leads to change. This creates a positive feedback loop in which reporting begets learning, and learning begets improvement [30]. Fourth, inter-professional collaboration should be actively promoted. Leadership should not be confined to physicians or administrators. Nurses, pharmacists, therapists, technicians, and clerical staff all bring unique perspectives and expertise. Supportive leadership embraces this diversity, creating spaces where all voices are heard and valued [31]. Finally, supportive leadership must be aligned with institutional goals and metrics. Accreditation standards, quality indicators, and strategic plans should reflect the centrality of leadership to patient safety. This alignment signals that supportive behavior is not optional or secondary but integral to success [32].

Beyond protocols: the standard range and relational guidance

In clinical practice, “the standard” is not a fixed point but a range, a flexible zone within which safe and appropriate care can be provided [33]. This concept is critical to understanding the true function of supportive leadership. Rather than rigidly enforcing protocol compliance, supportive leaders recognize that deviations may occasionally be justified based on patient context, clinical judgment, or resource availability. Their role is to help staff remain within this safe range, and when they veer toward its edges, to gently guide them back. This form of relational guidance, what some might call “nudging,” is neither punitive nor permissive. It acknowledges uncertainty and complexity while preserving accountability [34]. It requires a high level of mutual trust, in which staff feel safe acknowledging deviation and leaders feel confident offering direction without fear of alienation. Such leadership not only prevents harm but also cultivates adaptive learning, clinical maturity, and professional autonomy.

Psychological ownership and organizational vitality

Supportive leadership creates the conditions for what organizational psychologists call psychological ownership. This concept refers to the perception that one’s voice, actions, and values are not merely tolerated but indispensable to the organization’s identity and success [35]. Psychological ownership transforms employees from passive rule-followers into active co-creators. It embodies the difference between merely working in a hospital and truly belonging to it. Fostering this mindset requires more than verbal encouragement; it demands structural alignment. Staff must witness their suggestions not only being heard but also acted upon. Their insights should influence protocols, shape priorities, and inform policy. Recognition and reward systems must highlight initiative, integrity, and collaboration rather than mere compliance or tenure. When people sense that their contributions genuinely matter, they invest more of themselves, not out of obligation, but out of genuine commitment. Supportive leadership provides the fertile ground for such ownership to flourish. Leaders who listen with sincerity, validate contributions, and empower others enable individuals to invest both emotionally and intellectually in their work [36]. These leaders do not merely extract performance; they nurture purpose. Such investment, in turn, increases engagement, resilience, and collective responsibility, factors crucial to organizational vitality and patient safety [37].

The role of behavioral economics: nudge theory in practice

One of the most promising tools in the supportive leadership toolbox is nudge theory, derived from behavioral economics [38]. A “nudge” is a subtle change in the environment or presentation of choices that encourages better decisions without restricting freedom. In healthcare, where staff are often overloaded and fatigued, even small environmental cues can have a significant impact on behavior. Supportive leaders can use nudges to promote safety culture in unobtrusive yet powerful ways. For example, defaulting to generic prescribing options in electronic medical records can reduce medication errors. Posting peer compliance rates with hand hygiene or documentation standards can activate social norms. Rearranging physical spaces to encourage briefings and debriefings can foster reflection. Importantly, nudges are not manipulations; they are reinforcements of organizational values that help busy professionals act on their best intentions [39]. Leaders must be intentional in their use of nudges. They should be grounded in ethical transparency, aligned with patient safety goals, and evaluated for effectiveness. When done thoughtfully, nudges can reduce reliance on punitive controls and enhance autonomy, trust, and consistency [40].

Inclusive education and leadership development

Supportive leadership is inseparable from inclusive education. In a learning organization, diversity is not a challenge to be managed but a resource to be leveraged [41]. Inclusive leadership ensures that individuals from different backgrounds, experiences, and identities feel welcomed, respected, and supported in their learning journey.

This requires a systemic approach. Early detection systems should be in place to identify learners who are struggling, whether academically, socially, or emotionally. Individualized support plans, covering accommodations, mentorship, or mental health services, should be readily available. Faculty must be trained not only in teaching techniques but in cultural competence, trauma-informed care, and bias mitigation [42].

Inclusive education extends beyond learners to encompass staff at all levels. Leaders must cultivate workplaces in which all individuals, regardless of role, identity, or tenure, can develop their potential. This is not just about fairness. It is about safety. Diverse teams perform better, spot risks earlier, and generate more innovative solutions when they are psychologically safe [43].

Cross‑sector collaboration and the “knowledge ecosystem”

Modern healthcare does not exist in isolation. Hospitals are no longer just providers of care but hubs within a broader knowledge ecosystem that includes academia, community organizations, public health agencies, and patients themselves [44]. Supportive leadership must therefore transcend institutional boundaries. Leaders must model and facilitate collaboration across disciplines, sectors, and regions. They should encourage shared research agendas, interprofessional training, and joint quality improvement initiatives. They must support mechanisms, such as regional safety collaboratives, interdisciplinary rounds, or patient‑family advisory councils, that bring together multiple stakeholders [45]. This collaborative approach to leadership reflects a shift from vertical command to horizontal connection. It positions healthcare not as a siloed hierarchy but as a dynamic network, a place where shared knowledge leads to shared responsibility, and shared responsibility leads to better care [46].

Culture as strategy

Too often, organizational culture is seen as a byproduct of policies or the mood of senior leadership. In reality, culture is strategy. The values, assumptions, and behaviors that define how work is done are not incidental; they are determinative. Supportive leadership acknowledges this and works actively to shape culture in a positive direction [47]. This requires clarity of vision, consistency of behavior, and alignment of incentives. Supportive leaders must articulate the kind of culture they want to create, embody it in their daily interactions, and reinforce it through hiring, training, and performance evaluation. They must intervene when harmful subcultures, marked by blame, exclusion, or cynicism, emerge. And they must protect the time, space, and resources necessary for reflection, dialogue, and renewal [48]. Ultimately, culture is not changed through memos. It is changed through moments, moments when a leader listens deeply, responds graciously, or makes a difficult decision guided by values rather than expedience. Over time, these moments accumulate into a new organizational identity, one grounded in respect, resilience, and responsibility [49,50].

## Conclusions

Supportive leadership is not merely a management technique. It is a philosophy, a practice, and a promise. It recognizes that patient safety is not achieved through control, coercion, or compliance but through connection, care, and collaboration. It understands that excellence is not extracted from people but evoked through trust. To build safe, equitable, and sustainable healthcare systems, we must embrace leadership that listens before it directs, that asks before it answers, and that invites others to lead alongside. We must value people not only for what they produce but for who they are. We must move beyond blame and beyond fear, toward a future where safety is not a program or a project but a way of being. In this future, hospitals are not just buildings, and universities are not just institutions. They are communities of purpose, held together by leaders who understand that to support others is to lead them. To fully realize this vision, future work must integrate supportive leadership training into healthcare curricula, embed it into performance evaluations, and align it with regulatory and accreditation standards. Only by institutionalizing these values can healthcare systems ensure that supportive leadership becomes the norm rather than the exception.
